# Association of ATP‐binding cassette transporter genomic alterations and expressions with patient survival in breast and prostate cancer

**DOI:** 10.14814/phy2.70460

**Published:** 2025-07-10

**Authors:** Abdulaziz H. Alanazi, Nidhi Shenoy, Payaningal R. Somanath

**Affiliations:** ^1^ Clinical and Experimental Therapeutics, College of Pharmacy University of Georgia Augusta Georgia USA; ^2^ Department of Clinical Practice, College of Pharmacy Northern Border University Rafha Saudi Arabia

**Keywords:** ABC transporters, cancer, drug resistance, genetic alterations, patient survival

## Abstract

ATP‐Binding Cassette (ABC) transporters play a key role in drug resistance and cancer progression. We analyzed the correlation between ABC transporter gene alterations and patient survival in breast and prostate cancers using large‐scale genomic datasets. DepMap analysis revealed the overall dependency of cancer cell lines on ABC transporter genes such as ABCG2, ABCG1, ABCC4, ABCA2, ABCA3, ABCC2, ABCC3, ABCC6, ABCC7 (CFTR), and ABCC9, with ABCC6 and ABCC7 showing notably high dependence. Data from the cBioPortal for Cancer Genomics, incorporating multiple phase‐3 randomized clinical trials, identified genomic alterations including amplifications, deletions, and mutations in breast cancer patients (32 studies, 15,404 samples) and prostate cancer patients (29 studies, 13,857 samples). Kaplan–Meier analysis showed a significant correlation between ABC transporter gene alterations and overall survival in prostate cancer, but not in breast cancer. However, we found reduced relapse‐free survival correlating with reduced RNA expression in select ABC transporters in 4929 breast cancer patients in a KMplot analysis. These findings highlight the potential role of ABC transporters in prostate and breast cancer prognosis and warrant further investigation into their therapeutic implications.

## 1 INTRODUCTION

Cancer remains one of the leading causes of mortality worldwide, with breast and prostate cancer being among the most prevalent malignancies in women and men, respectively (Siegel et al., 2025). These cancers, along with others such as lung and colorectal cancer, contribute significantly to the global cancer burden, necessitating continued efforts to improve early detection, treatment strategies, and patient outcomes. Despite remarkable advancements in cancer therapeutics, including chemotherapy, targeted therapies, and immunotherapy, treatment resistance remains a major challenge that limits the efficacy of these interventions and contributes to disease progression and relapse (Ingham et al., 2025). One of the key mechanisms underlying resistance to chemotherapy and targeted therapies is the activity of ATP‐binding cassette (ABC) transporters (Pote & Gacche, 2023). These membrane‐bound proteins play a crucial role in cellular homeostasis by actively transporting a wide range of substrates across biological membranes. In cancer cells, ABC transporters function as efflux pumps that expel chemotherapeutic agents and other cytotoxic compounds from the cells, thereby reducing intracellular drug accumulation. This process diminishes the effectiveness of anticancer drugs, leading to multidrug resistance (MDR), a phenomenon in which cancer cells become resistant to multiple structurally and mechanistically distinct drugs. MDR remains a significant obstacle in cancer treatment, often resulting in therapeutic failure and poor prognosis for patients (Pote & Gacche, 2023).

ABC transporters are encoded by a large family of genes that include subfamilies such as ABCG, ABCC, and ABCA (Pasello et al., 2020). Several ABC transporter family members, including P‐glycoprotein (P‐gp, also known as ABCB1), multidrug resistance‐associated proteins (MRPs), and breast cancer resistance protein (BCRP, also known as ABCG2), have been extensively studied for their role in drug resistance (Goebel et al., 2021). These transporters are often overexpressed (Kadioglu et al., 2020; Moore et al., 2023) or downregulated (Demidenko et al., 2015) in many cancers following prolonged exposure to chemotherapeutic agents, further exacerbating treatment resistance. Given their pivotal role in MDR (Xiao et al., 2021), targeting ABC transporters has been an area of intense research, with strategies focusing on the development of inhibitors, alternative drug delivery systems, and combination therapies to overcome resistance and enhance treatment efficacy. However, a comprehensive, large‐scale analysis of ABC transporter gene alterations and their potential link with patient survival in cancer, including breast and prostate cancer, remains lacking. To address this gap, we conducted a systematic investigation of ABC transporter gene alterations in breast and prostate cancer using publicly available genomic datasets from cBioPortal for Cancer Genomics. Our study analyzed data from multiple randomized, multicentered phase‐3 clinical trials, encompassing thousands of patient samples. We assessed the frequency of genomic alterations in key ABC transporter genes, including ABCG2, ABCG1, ABCC4, ABCA2, ABCA3, ABCC2, ABCC3, ABCC6, ABCC7 (CFTR), and ABCC9. Additionally, we performed a survival analysis using Kaplan–Meier plots to determine the potential prognostic significance of these alterations in both cancer types.

Our findings reveal distinct patterns of ABC transporter gene alterations between breast and prostate cancer. While genomic alterations were prevalent in both malignancies, significant correlations between ABC transporter gene alterations and patient survival were observed exclusively in prostate cancer, whereas no such correlation on overall survival was found in breast cancer. However, KMplot analysis of a TCGA study involving 4929 breast cancer patients revealed a correlation between decreased RNA expressions of select ABC transporters and reduced relapse‐free survival. These results suggest that ABC transporters play a crucial role in prostate and breast cancer progression, highlighting the need for further functional validation with larger datasets and the exploration of targeted therapeutic strategies.

## 2 MATERIALS AND METHODS

### 2.1 DepMap analysis of genetic mutations in ABC transporter genes and their dependency across cancer cells

The Cancer Dependency Map (DepMap; https://depmap.org/portal/) by the Broad Institute, which analyses thousands of cancer cell lines to determine gene dependencies using RNA interference (RNAi) and CRISPR‐Cas9 knockout techniques (McFarland et al., 2018) was used in our study. Within the DepMap database, the ATA (Area‐Under‐the‐Threshold Activity) score serves as a key metric for evaluating whether a specific cell line relies on a particular gene. This score integrates gene dependency data from CRISPR and RNAi screens to measure the impact of gene depletion on cell viability. A more negative ATA score indicates a stronger dependency, with defined thresholds (e.g., −0.5 for CRISPR screens) used to differentiate essential genes from non‐essential ones. Scores approaching zero or positive values suggest that the gene is not crucial for the survival of the cell line. This classification is instrumental in identifying potential therapeutic targets by highlighting genes that are selectively essential in certain cancer types. Dependency scores, which range from 0 to −1 (Gene effect), indicate the probability that a gene is vital for tumor cell survival, with lower values signifying greater dependency. The DEMETER2 and Chronos algorithms analyze RNAi and CRISPR data, respectively, and their results are represented as bell‐curve distributions. Each gene was assessed based on its impact on cell viability, the proportion of dependent cell lines, and its classification as “strongly selective” (indicating essentiality in specific cancers). The “strongly selective” classification in DepMap is based on statistical modeling of dependency score distributions across all cell lines, which can reveal selective patterns, such as bimodal or skewed distributions, even when dramatic dropout is not visually apparent. This analysis provides valuable insights into key genes for potential therapeutic intervention. In our study, we examined tumor cell line dependencies on individual ABC transporter genes (ABCG2, ABCG1, ABCC4, ABCA2, ABCA3, ABCC2, ABCC3, ABCC6, ABCC7/CFTR, and ABCC9) using the latest DepMap dataset. We utilized CRISPR (Avana) Public 19Q2 and Combined RNAi data (Broad, Novartis, and Marcotte) to gain a comprehensive understanding of gene dependencies, enabling the identification of cancer vulnerabilities linked to ABC transporters.

### 2.2 cBioportal data analysis of genomic alterations in ABC transporter genes in breast and prostate cancers

To investigate genetic alterations in ABC transporter genes within clinical tumor samples, we utilized the cBioPortal for Cancer Genomics database (https://www.cbioportal.org/) (Cerami et al., 2012; Ping et al., 2014). This platform enabled the analysis of both DNA‐level modifications and RNA‐level gene expression. In prostate cancer, the analysis was carried out from 29 curated transcriptomic studies from the Cancer Genome Atlas (TCGA), comprising 13,387 profiled samples, while breast cancer analysis was performed from 32 studies involving a sample size of 15,404. A detailed list of Cancer subtypes/stages of breast and prostate cancers included in the CBioPortal data analysis is presented in the Supplemental Table 1. These studies were carefully selected to exclude pediatric cases and duplicate samples, ensuring the robustness of the analysis. Gene expression at the RNA level and mutation data were queried across various cancer types using the TCGA dataset. To visualize ABC transporter alterations, an oncoprint was generated, highlighting genetic changes in breast and prostate cancers, as well as summarizing alterations across all cancer types. Prostate and breast cancer samples were classified into four distinct subgroups each, based on the presence of structural variants, mutations, and copy number alterations (CNAs) as defined in cBioPortal. For prostate cancer, the subgroups included the following: “Prostate,” representing noninvasive tumors characterized by structural variants, mutations, and CNAs; “Prostate Cancer,” encompassing invasive cases assessed with the same genomic data types; “Prostate Adenocarcinoma,” classified using mutation and CNA data only; and “Prostate Cancer, Not Otherwise Specified (NOS),” identified solely based on mutation data. Similarly, breast cancer samples were grouped as follows: “Breast,” comprising noninvasive tumors analyzed using mutation data alone; “Breast Cancer,” representing noninvasive cases characterized by mutations, structural variants, and CNAs; “Breast Sarcoma,” defined by mutation and CNA data; and “Invasive Breast Sarcoma,” which included invasive tumors with alterations identified across mutations, CNAs, and structural variants. A more detailed classification of samples on general genetic alterations in breast cancer and prostate cancer are provided in supplemental Figure 1 and Supplemental Figure 2, respectively.

### 2.3 Analysis of the correlation between ABC transporter gene expression and overall survival in breast and prostate cancer patients

Survival data from TCGA datasets available on cBioPortal were extracted and systematically organized in an Excel spreadsheet, distinguishing censored cases from deceased patients. Patient groups with ABC transporter alterations were compared against those without such changes. Kaplan–Meier survival curves were then constructed using SPSS software, following previously established methods (Vo et al., 2025).

Specifically for breast cancer, survival analysis was also conducted using the Pan‐Cancer datasets available on the www.kmplot.com online tool (Gyorffy, 2021). These datasets are derived from TCGA study generated using the Illumina HiSeq 2000 platform, with survival information obtained from a previous publication (Liu et al., 2018). Redundant samples were removed, biased arrays were excluded for quality control, and the proportional hazards assumption was verified. Kaplan–Meier survival plots were generated by the KMplot software. Relapse‐free survival of breast cancer patients with the RNA expression changes in ABC transporter genes was performed from a total of 4929 patients using the inbuilt software in the KMplot database.

In the Kaplan–Meier survival plots, “altered” refers to patients whose tumors have at least one genomic alteration in ABC transporter genes; this includes amplifications (increased gene copy number), deletions (loss of gene copies), or mutations (changes in the DNA sequence) of these genes. “Unaltered” refers to patients whose tumors do not have any such genomic changes in the ABC transporter genes, meaning the genes are in their typical, unmodified state. These groupings help compare survival outcomes between patients with and without ABC transporter gene dysregulation.

### 2.4 Statistical analysis

Statistical analyses were conducted using SPSS v29 and GraphPad Prism v11. Median and mean survival times, hazard ratios, and log‐rank tests were utilized to evaluate the prognostic significance of ABC transporter alterations (Vo et al., 2025).

## 3 RESULTS

### 3.1 Analysis of tumor cell line dependency on ABC transporters

**FIGURE 1:**
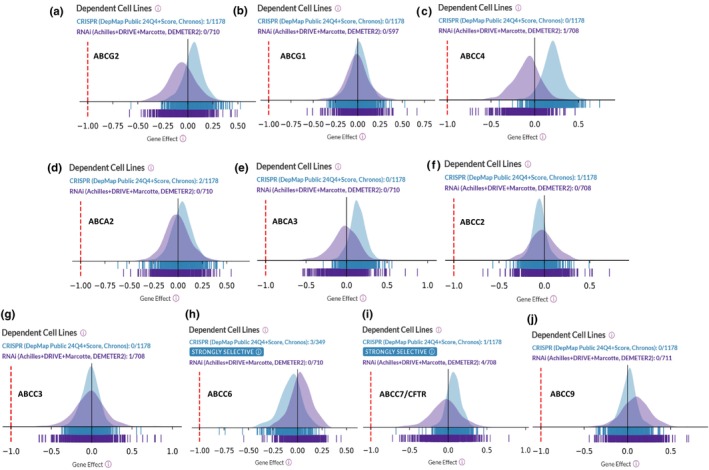
(a–j) Analysis of cancer cell line dependency on selected ABC transporter genes using data from the DepMap CRISPR (blue) and RNAi (purple) screening datasets. Each panel displays the distribution of dependency scores (x‐axis: Gene Effect) for a specific ABC gene across a panel of tumor cell lines. The Gene Effect score quantifies the impact of gene suppression (via CRISPR or RNAi) on cell viability: A score of 0 indicates no effect, while more negative values reflect increased dependency, that is, stronger loss of cell viability upon gene knockdown or knockout. A score of −1 is approximately the median effect observed for established essential genes. This analysis highlights gene‐specific vulnerabilities across diverse cancer cell lines.

**FIGURE 2:**
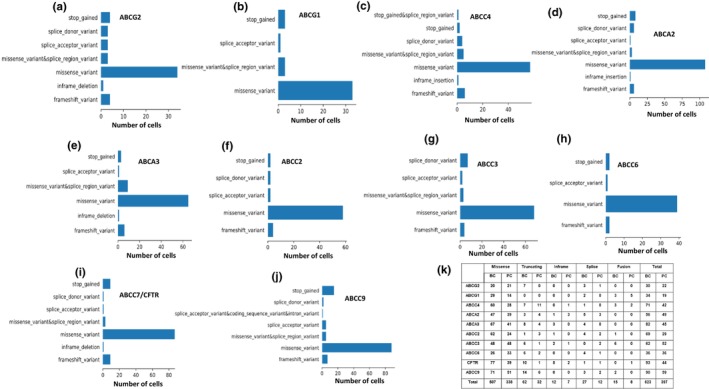
(a–j) Bar graphs showing the distribution and frequency of mutation types, including missense, nonsense, frameshift, and splice site mutations, in individual ABC transporter genes across a wide range of cancer cell lines. Data are derived from the DepMap portal, which aggregates large‐scale genomic profiling of cell lines. Each panel corresponds to a different ABC gene, highlighting the spectrum of observed mutations. (k) Summary chart of mutation types and their relative frequencies in ABC transporter genes specifically within breast and prostate cancer patient samples, based on cBioPortal data. This comparison between tumor tissues and cell lines provides insight into potential tissue‐specific mutation patterns.

Our data provides a comprehensive comparison of DepMap dependency scores for ABCG2, ABCG1, ABCC4, ABCA2, ABCA3, ABCC2, ABCC3, ABCC6, ABCC7/CFTR, and ABCC9, based on CRISPR analysis of 1178 tumor cell lines and RNAi analysis of 711 cell lines (Figure 1a–j). The cancer cell lines were not subjected to any treatments, and no data are available regarding potential mutations in any cell population. The “Gene Effect” (x‐axis) is the CRISPR‐derived dependency score, where lower (more negative) values indicate greater loss of cell viability; 0 means no effect, and −1 approximates the median impact of essential gene knockouts. Notably, in the majority of ABC transporters, RNAi analysis revealed a higher fraction of dependent cell lines compared to CRISPR. ABCC6 and ABCC7 were identified as strongly selective genes, underscoring their potential as cancer‐specific vulnerabilities (Figure 1). DepMap analysis also revealed the type of mutations and the percentage of cancer cells (all types combined) with each type of mutation in various ABC transporter genes. Missense mutations were the most common among all the ABC transporters analyzed (Figure 2a–k). Other types of mutations observed in ABC transporter genes across all members include splice acceptor or donor variants and frameshift mutations (Figure 2). Figure 2k shows the types, frequencies, and combined numbers of mutations in individual ABC transporter genes in breast and prostate cancer patients.

### 3.2 Clinical analysis of alterations and expressions in ABC transporter genes in breast and prostate cancer

**FIGURE 3:**
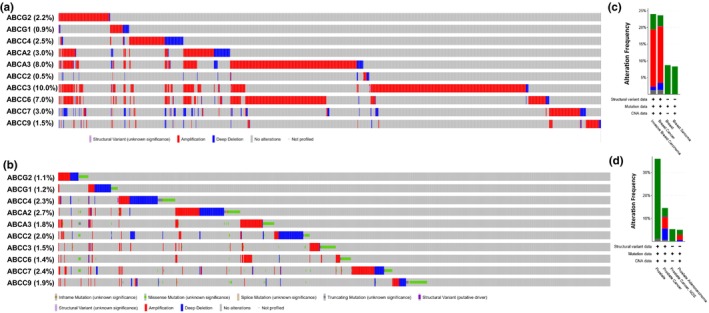
(a and b) Oncoprints from cBioPortal showing genomic alterations in ten ABC transporter genes (ABCG2, ABCG1, ABCC4, ABCA2, ABCA3, ABCC2, ABCC3, ABCC6, ABCC7/CFTR, and ABCC9) across 25 breast cancer and 29 prostate cancer studies. Only samples with at least one altered gene are shown as colinear vertical lines; the majority of samples, with no alterations in these genes, are not displayed. Alteration types are color‐coded: Amplification (red), deep deletion (blue), and point mutation (green). (c and d) Bar graphs summarizing the frequency of these alterations across the same cohorts.

In the second phase of our study, we leveraged cBioPortal, a widely used open‐source cancer genomics database originally developed by Memorial Sloan Kettering Cancer Center (Cerami et al., 2012; Ping et al., 2014), to conduct a comprehensive analysis of genomic alterations in ABC transporter genes across a diverse range of cancer subtypes. This database provides an extensive collection of curated genomic data, enabling researchers to explore alterations in key genes across various tumor types. To assess the prevalence and distribution of these genomic changes, we performed a systematic query across 32 curated studies, encompassing a total of 15,404 breast tumor samples and 29 curated studies, encompassing a total of 13,387 prostate tumor samples. This large‐scale dataset allowed us to capture a broad spectrum of genetic alterations affecting ABC transporter genes, offering valuable insights into their potential roles in breast (Figure 3a) and prostate cancer (Figure 3b) progression. The identified genomic alterations were categorized into distinct types, including mutations (green), fusions (purple), amplifications (red), deep deletions (blue), and multiple alterations (gray). These classifications provided a detailed view of the genetic landscape of ABC transporter genes in breast and prostate cancers. The results were visualized to facilitate interpretation and highlight patterns of genetic variation (Figure 3a,b).

**FIGURE 4:**
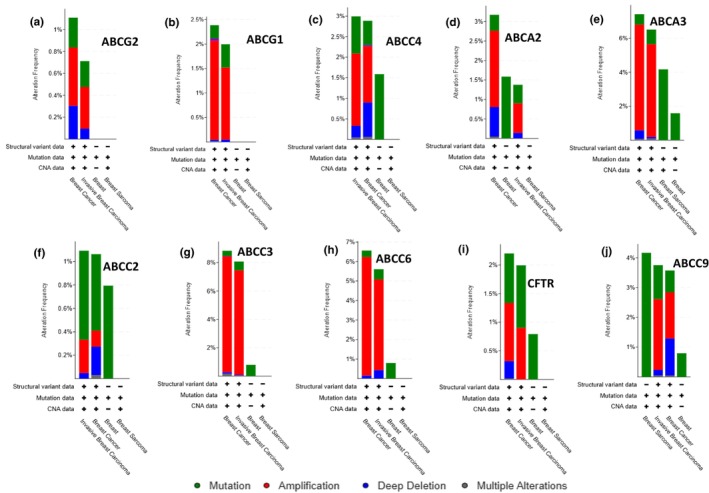
(a–j) Bar graphs showing the frequency of genomic alterations (gene amplifications, deep deletions, and mutations) for individual ABC transporter genes across 15,404 breast cancer samples from 32 clinical studies in cBioPortal. Each panel corresponds to a different ABC gene, depicting the proportion of tumors with each alteration type. “Altered” refers to samples with at least one amplification, deletion, or mutation in the gene; “unaltered” indicates no detectable genomic changes. This dataset provides a broad overview of alteration patterns across breast cancer cohorts, highlighting ABC transporters potentially involved in disease progression or therapeutic response.

**FIGURE 5:**
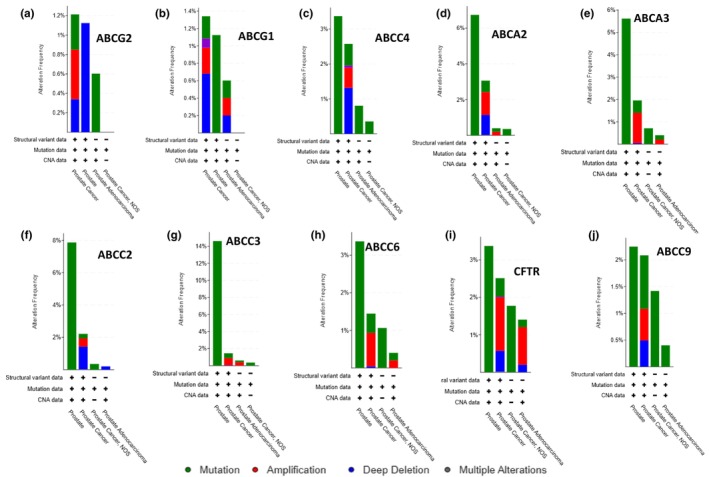
(a–j) Bar graphs illustrating the frequency of genomic alterations (amplifications, deep deletions, and mutations) in individual ABC transporter genes across 13,857 prostate cancer samples from 29 clinical studies in cBioPortal. Each panel represents a different ABC gene, showing the proportion of tumors with specific alterations. “Altered” refers to samples with at least one genomic change in the gene, while “unaltered” indicates no detected alterations. This analysis highlights recurrently affected transporters that may play roles in prostate cancer biology or therapeutic response.

In our analysis, gene amplification was the predominant genetic alteration observed in breast cancer tissues (Figure 3c), whereas prostate cancer tissues exhibited a higher frequency of mutations compared to other types of genetic alterations (Figure 3d). Interestingly, the data revealed a higher frequency of missense mutations in control prostate tissue compared to prostate cancer, a surprising observation that may reflect biological variability or technical and sampling differences. The overall frequency of genetic alterations in breast cancer ranged from 1% in ABCG2 to 9% in ABCC3 (Figure 3a). Similarly, in prostate cancer, the alteration frequency varied from 1.1% in ABCG2 to 2.7% in ABCA2 (Figure 3b), with ABCG2 consistently showing the lowest level of genetic alterations in both cancer types. Deep deletions were significantly more prevalent in prostate cancer tissues (Figure 3b) compared to breast cancer tissues, where they were relatively rare and limited to ABCG2, ABCC4, ABCA2, ABCC2, and ABCC9 (Figures 3a and 4). In contrast, prostate cancer tissues exhibited frequent deep deletions in ABCG2, ABCG1, ABCC4, ABCA2, ABCC2, ABCC7/CFTR, and ABCC9 (Figures 3b and 5). Regarding mutation frequency, the highest mutation rates in breast cancer tissues were observed in ABCC2 and ABCC9. In prostate cancer tissues, however, gene amplifications and deep deletions were most frequently detected in ABCG2 and ABCG1 (Figure 5a,b). These findings highlight specific ABC transporter genes as recurrently altered in breast and prostate cancers, suggesting their potential as biomarkers and therapeutic targets.

### 3.3 Analysis of survival curves for patients with alterations in ABC transporter genes in breast and prostate cancer

**FIGURE 6:**
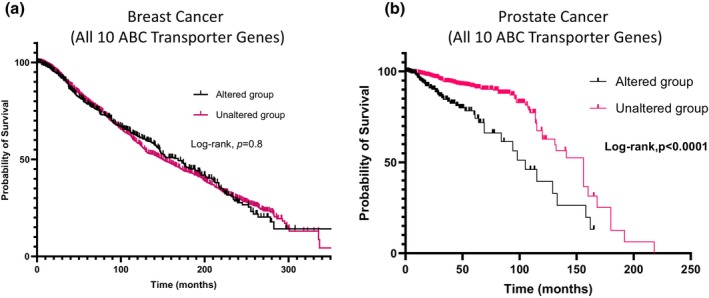
(a and b) Kaplan–Meier survival plots showing the association between combined genomic alterations in ABC transporter genes (amplifications, deletions, and mutations) and overall survival in breast cancer (a) and prostate cancer (b) cohorts. Data are obtained from cBioPortal, with patients stratified into “altered” (tumors harboring at least one ABC gene alteration) and “unaltered” (no alterations) groups. These plots illustrate the potential prognostic impact of ABC transporter gene dysregulation on patient survival over time.

Next, we investigated the correlation between ABC transporter gene alterations and patient survival in breast and prostate cancers. Using cBioPortal data, we performed Kaplan–Meier survival analyses with SPSS Statistics to compare overall survival between patients with altered ABC transporter genes and those with unaltered expressions. The survival curves illustrate the probability of survival over time (in months), comparing patients with genetic alterations (blue) to those without alterations (black) in breast cancer, and patients with alterations (green) to those without alterations (red) in prostate cancer (Figure 6). Our analysis revealed a significant reduction in overall survival probability for prostate cancer patients with ABC transporter gene alterations (all member genes combined) (Figure 6b). In contrast, survival outcomes in breast cancer patients did not significantly differ between the altered and unaltered groups (Figure 6a). These findings underscore the potential impact of ABC transporter gene alterations on patient prognosis, particularly in prostate cancer, and highlight the need for further investigation into their role in cancer progression and therapeutic resistance.

**FIGURE 7:**
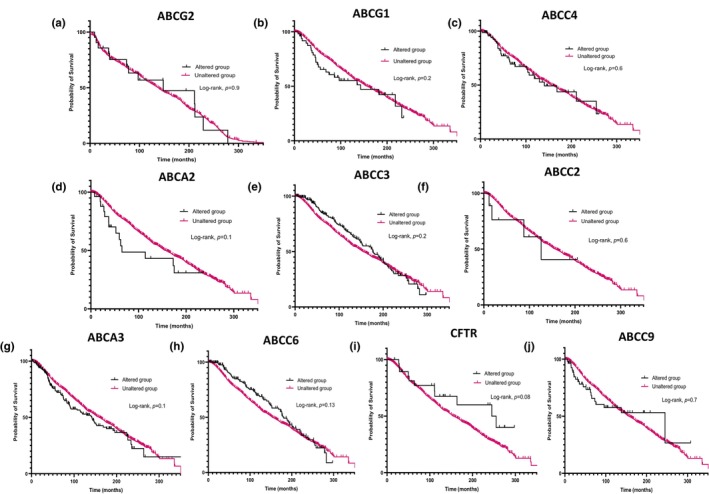
(a–j) Kaplan–Meier survival plots assessing the association between alterations in individual ABC transporter genes and overall survival in breast cancer patients. Each panel corresponds to a specific gene and compares survival between patients with (“altered”) and without (“unaltered”) amplifications, deletions, or mutations in that gene. Data are obtained from cBioPortal using clinically annotated breast cancer cohorts. These analyses highlight the potential prognostic relevance of specific ABC transporter gene alterations.

**FIGURE 8:**
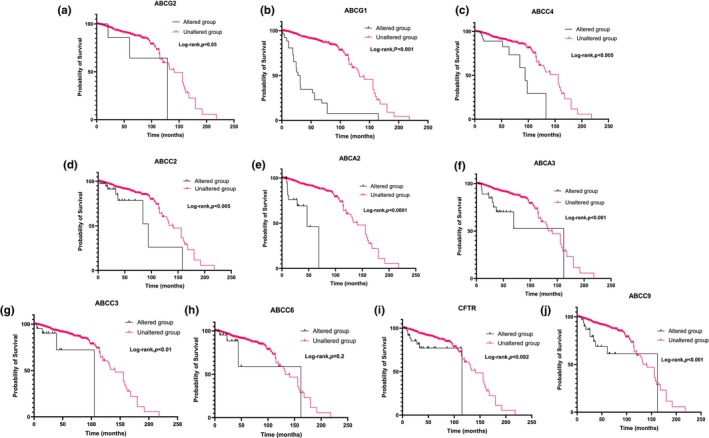
(a–j) Kaplan–Meier survival plots showing the association between alterations in individual ABC transporter genes and overall survival in prostate cancer patients. Each panel compares survival between patients with (“altered”) and without (“unaltered”) amplifications, deletions, or mutations in the indicated gene. Data are sourced from cBioPortal using aggregated clinical datasets from multiple prostate cancer cohorts. These plots evaluate the potential prognostic significance of individual ABC gene alterations in prostate cancer.

Analysis of individual ABC transporter genes in breast cancer tissues revealed no significant correlation between genetic alterations in any of the 10 ABC transporter genes analyzed and patient survival (Figure 7). However, an interesting, though nonsignificant, trend suggested that patients with ABCC7/CFTR genetic alterations may have improved survival outcomes (Figure 7i), warranting further investigation into its potential protective role. In contrast, analysis of prostate cancer tissues revealed a notable association between genetic alterations in multiple ABC transporter genes and reduced patient survival. Specifically, alterations in ABCG2, ABCG1, ABCC4, ABCC2, ABCA2, ABCA3, ABCC3, ABCC7/CFTR, and ABCC9 were linked to poorer survival outcomes (Figure 8). The only exception was ABCC6, which did not exhibit a significant correlation with patient survival (Figure 8h). These findings suggest that specific ABC transporter gene alterations may play a more critical role in prostate cancer prognosis compared to breast cancer, highlighting their potential as biomarkers and therapeutic targets for disease management.

**FIGURE 9:**
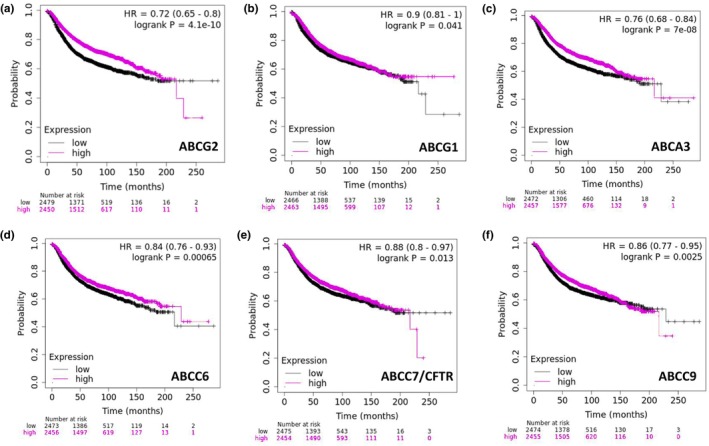
(a–f) Kaplan–Meier plots showing relapse‐free survival (RFS) in breast cancer patients stratified by RNA expression alterations in selected ABC transporter genes (ABCG2, ABCG1, ABCA3, ABCC6, ABCC7/CFTR, and ABCC9). Patients were grouped based on differential gene expressions, with data and clinical outcomes obtained from cBioPortal. These genes showed statistically significant associations with RFS, suggesting a potential role in disease recurrence. “Altered” indicates tumors with expression changes linked to underlying amplifications, deletions, or mutations; “unaltered” refers to tumors without such genomic changes.

The KMplot survival analysis of breast cancer patients with RNA expression changes in ABC transporters indicated no significant differences in overall survival. However, RNA expression alterations in six different ABC transporters were found to be associated with reduced relapse‐free survival (RFS) in patients exhibiting altered RNA expression levels, suggesting that dysregulation of these transporters may contribute to disease recurrence despite no apparent effect on overall survival (Figure 9a–f). Notably, no data on prostate cancer patients were available in the KMplot database, limiting the scope of the analysis for this cancer type.

## 4 DISCUSSION

ABC transporters have been linked to various human diseases and are considered potential targets for small‐molecule drugs (Moore et al., 2023), particularly in cancer (Muriithi et al., 2020), where they are believed to play a crucial role in the development of drug resistance (Duvivier et al., 2024; Xiao et al., 2021). Our study offers an in‐depth analysis of the dependencies of ABC transporter genes across various cancer cell lines, and explores their alterations and clinical significance in breast and prostate cancers. By integrating DepMap dependency scores, genomic alteration data from cBioPortal, and patient survival analyses, we have identified distinct patterns in ABC transporter gene involvement in these cancers, highlighting their potential as biomarkers and therapeutic targets. DepMap analysis revealed variations in ABC transporter gene dependencies across a broad spectrum of tumor cell lines, highlighting their differential roles in cancer cell survival beyond their perceived role in developing chemotherapeutic drug resistance (Muriithi et al., 2020). Notably, ABCC6 and ABCC7/CFTR emerged as strongly selective genes. Both ABCC6 (Aranyi et al., 2013) and ABCC7/CFTR (Bhattacharya et al., 2022; Kovacova et al., 2024) have also been shown by others to harbor mutations that impair function and lead to disease, suggesting they may serve as cancer‐specific vulnerabilities. Interestingly, RNAi analyses identified a greater fraction of dependent cell lines compared to CRISPR‐based analyses, suggesting that partial gene knockdown may reveal additional functional dependencies that are not as evident in complete knockout models. This difference likely reflects several key distinctions between the two technologies. For instance, RNAi often results in incomplete suppression of gene expression, which may allow cells to survive with reduced, but not absent, protein function, thereby identifying genes where only partial activity is sufficient for viability. In contrast, CRISPR‐mediated gene editing typically results in complete gene knockout, which may unmask only those dependencies where total loss of function is lethal. Furthermore, differences may also arise from technical artifacts. In particular, CRISPR targeting of highly amplified genomic regions, such as amplified ABC transporter genes, can induce hypersensitivity due to the accumulation of numerous double‐stranded DNA breaks, overwhelming the cellular DNA repair capacity (Di Carlo & Sorrentino, 2024). This can lead to toxicity that does not reflect genuine gene dependency but rather the structural vulnerability of the genome. These considerations underscore the importance of interpreting functional dependency data in the context of the specific characteristics and limitations of each perturbation platform.

In addition to dependency analysis, we characterized the mutational landscape of ABC transporter genes across cancer cell lines. Gene amplifications and missense mutations were the most common types of alterations observed, followed by splice site and frameshift mutations. These mutational patterns and gene expression changes may contribute to altered protein function, potentially impacting drug transport and resistance mechanisms in various cancers, as have been demonstrated by others to affect cancer patient outcomes (Kadioglu et al., 2020), including breast cancer (Hlavac et al., 2020). Through a large‐scale analysis of 13,387 and 15,404 prostate and breast tumor samples, we identified distinct patterns of ABC transporter gene alterations between the two cancer types. Gene amplification was the predominant alteration in breast cancer, whereas prostate cancer exhibited a higher frequency of mutations. ABCG2 consistently displayed the lowest level of genetic alterations in both cancers, suggesting a potential role in maintaining cellular homeostasis. Interestingly, in a non‐small cell lung cancer study, ABCG2 was expressed at low levels in NSCLC with no correlation to lung cancer aggressiveness, yet higher expression was associated with improved overall survival (Jelen et al., 2024), suggesting that despite its low expression and genetic variability, ABCG2 plays a crucial role in lung cancer patient survival and may influence the effectiveness of cancer therapy. The significance of targeting ABCG2 (also known as breast cancer resistance protein/BCRP) in breast cancer (Zattoni et al., 2022) and prostate cancer (Sabnis et al., 2017) also been reported.

Deep deletions were significantly more prevalent in prostate cancer than in breast cancer, with frequent occurrences in ABCG2, ABCG1, ABCC4, ABCA2, ABCC2, ABCC7/CFTR, and ABCC9. This correlates with a report on frequent downregulation of the ABC transporter genes in prostate cancer (Demidenko et al., 2015). In contrast, breast cancer exhibited relatively lower deep deletion rates, restricted to ABCG2, ABCC4, ABCA2, ABCC2, and ABCC9. These findings suggest that prostate cancer may rely on specific ABC transporter genes for tumor progression, whereas breast cancer may employ alternative mechanisms of adaptation. Nevertheless, the downregulation of ABCA10, a gene that we did not investigate in the current study, has been reported as a prognostic marker associated with immune infiltration in breast cancer (Chu et al., 2022), suggesting that the fate of breast cancer progression and/or drug resistance is not solely dependent on the ABC transporter molecules investigated in the current study.

Survival analysis revealed a significant association between ABC transporter gene alterations and overall survival in prostate cancer, whereas no such correlation was observed in breast cancer. Specifically, prostate cancer patients with genetic alterations in ABC transporter genes exhibited a marked reduction in survival probability, suggesting that these genes may play a critical role in disease progression and patient prognosis. In contrast, breast cancer patients did not show significant differences in survival based on ABC transporter gene alterations. Analysis of individual genes further supported the clinical significance of ABC transporter gene alterations in prostate cancer. Alterations in ABCG2, ABCG1, ABCC4, ABCC2, ABCA2, ABCA3, ABCC3, ABCC7/CFTR, and ABCC9 were all associated with reduced survival, whereas ABCC6 appeared to have no significant prognostic impact. Interestingly, in breast cancer, while no statistically significant associations were observed, a potential trend toward improved survival was noted in patients with ABCC7/CFTR alterations. This observation contrasts with the findings of Kovácová et al. (2024), who reported that ABCC7 alterations were linked to poorer outcomes. This discrepancy underscores the need for further research to clarify the role of ABCC7 in breast cancer prognosis (Kovacova et al., 2024). However, further analysis of relapse‐free survival (RFS) revealed that six ABC transporters were significantly associated with reduced RFS in patients exhibiting altered mRNA expression levels. This suggests that dysregulation of these transporters may contribute to disease recurrence despite the lack of a significant impact on overall survival. The potential role of these transporters in promoting resistance mechanisms or facilitating metastatic processes warrants further investigation. Understanding how these transporters influence recurrence may provide valuable insights into breast cancer progression and potential therapeutic targets.

The distinct alteration patterns observed in breast and prostate cancers suggest that ABC transporter genes contribute differently to tumorigenesis and progression in these malignancies. The strong association between ABC transporter gene alterations and poor survival in prostate cancer underscores their potential as prognostic biomarkers. Additionally, the identification of ABCC6 and ABCC7/CFTR as selective vulnerabilities in DepMap analyses suggests that these genes may serve as promising therapeutic targets. Further research is needed to elucidate the functional consequences of these genetic alterations and their potential roles in drug resistance. Given the well‐established involvement of ABC transporters in multidrug resistance, understanding how these genes influence therapeutic response could lead to novel strategies for overcoming treatment resistance in cancer patients.

Despite the valuable insights provided by our study, several limitations should be considered. First, the focus on breast and prostate cancers may limit the generalizability of our findings to other cancer types, as different subtypes may exhibit distinct patterns of ABC transporter gene alterations. Additionally, the cross‐sectional nature of the data from cBioPortal restricts our ability to establish causal relationships between gene alterations and cancer progression. Another limitation is the potential bias toward early‐stage cancers in the datasets, which may obscure transporter roles in late‐stage outcomes. Potential biases in sample representation and the exclusion of certain genetic alterations, such as epigenetic changes, may also affect the results. While survival analyses suggest a correlation between ABC transporter alterations and patient outcomes, confounding factors like prior treatments or comorbidities were not fully accounted for. Functional validation experiments and additional studies linking genetic alterations to therapeutic responses are needed to further validate these findings. Finally, the reliance on a single database (cBioPortal) underscores the need for independent validation to confirm the robustness and reproducibility of our results. Another limitation of our study is the unavailability of prostate cancer patient data in the KMplot database, which restricted our ability to extend the analysis to this cancer type. Future studies incorporating additional datasets and independent validation cohorts would be necessary to determine whether similar trends exist in prostate cancer or other malignancies.

In summary, our study highlights the significance of ABC transporter gene alterations in breast and prostate cancers and their potential impact on patient prognosis. The distinct patterns of genetic alterations and survival outcomes between the two cancer types suggest that the ABC transporter genes we investigated play a more prominent role in prostate cancer progression. These findings provide a foundation for future research aimed at targeting ABC transporters in cancer therapy and improving patient outcomes.

## FUNDING INFORMATION

The study was supported by the Kenneth L. Waters Foundation Endowment and National Institutes of Health grant UL1TR002378 to PRS.

## CONFLICT OF INTEREST STATEMENT

The authors declare no conflict of interest.

## ETHICS APPROVAL AND CONSENT TO PARTICIPATE

This study was conducted using the publicly available datasets, thus exempted from IRB approvals.

## Supporting information


Data S1.

## Data Availability

The datasets used and/or analyzed in the current study are available from the corresponding author upon reasonable request.
