# Supplementary material for: Association of ATP‐binding cassette transporter genomic alterations and expressions with patient survival in breast and prostate cancer
Source: Physiol Rep. 2025 Jul 10;13(13):e70460. doi: 10.14814/phy2.70460 (PMC12245978; doi:10.14814/phy2.70460)
Supplement: Supplementary file 1 — Data S1. [file PHY2-13-e70460-s001.pdf]

Supplemental Data

Association of ATP-binding cassette transporter genomic alterations and expressions with patient survival in breast and prostate cancer

Abdulaziz H. Alanazi, Nidhi Shenoy, and Payaningal R. Somanath

Supplemental Table 1: Cancer subtypes/stages of breast and prostate cancers that were included in the CBioPortal data analysis.

#	**Breast Cancer Subtypes**	**Number of Studies**
1	Breast cancer (subtypes not specified)	3
2	Metastatic breast carcinoma	6
3	Non-CDH1 Invasive Lobular carcinoma	1
4	Invasive breast carcinoma	20
5	Adenoid cystic carcinoma	1
6	Metaplastic breast cancer	1

**#**	**Prostate Cancer Subtypes**	**Number of Studies**
1	Prostate cancer (subtypes not specified)	7
2	Prostate Adenocarcinoma	15
3	Metastatic Prostate Adenocarcinoma	5
4	Castration-Resistant Prostate Cancer	1
5	Prostate Neuroendocrine Carcinoma	1

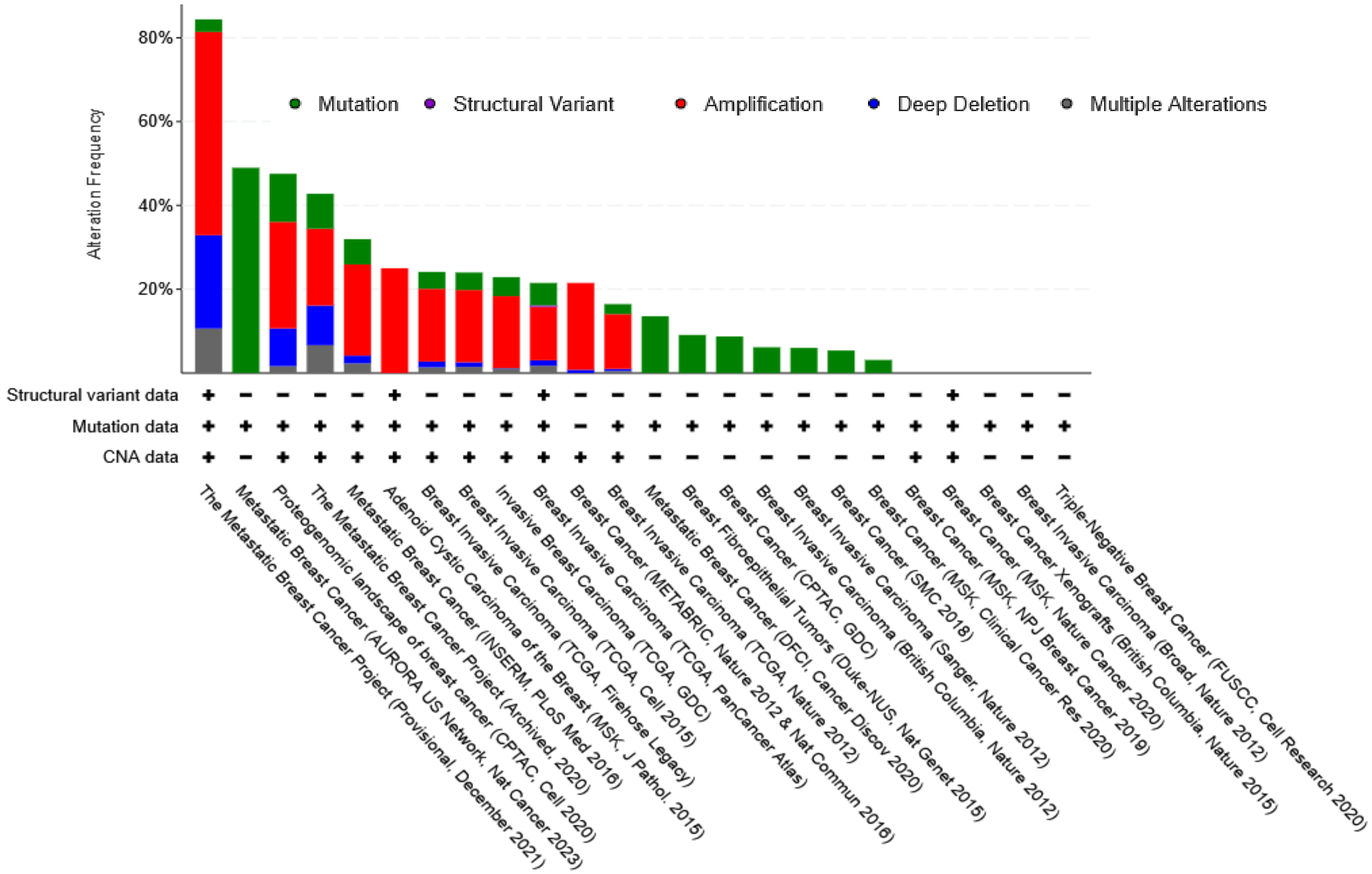

Supplemental Figure 1: Bar graph showing the frequency of genomic alterations, including gene amplifications, structural variants, deep deletions, and mutations, for ABC transporter genes across various breast cancer patient cohorts available in cBioPortal from 15404 patients in 32 studies. Data Link: https://bit.ly/3FCWg5m

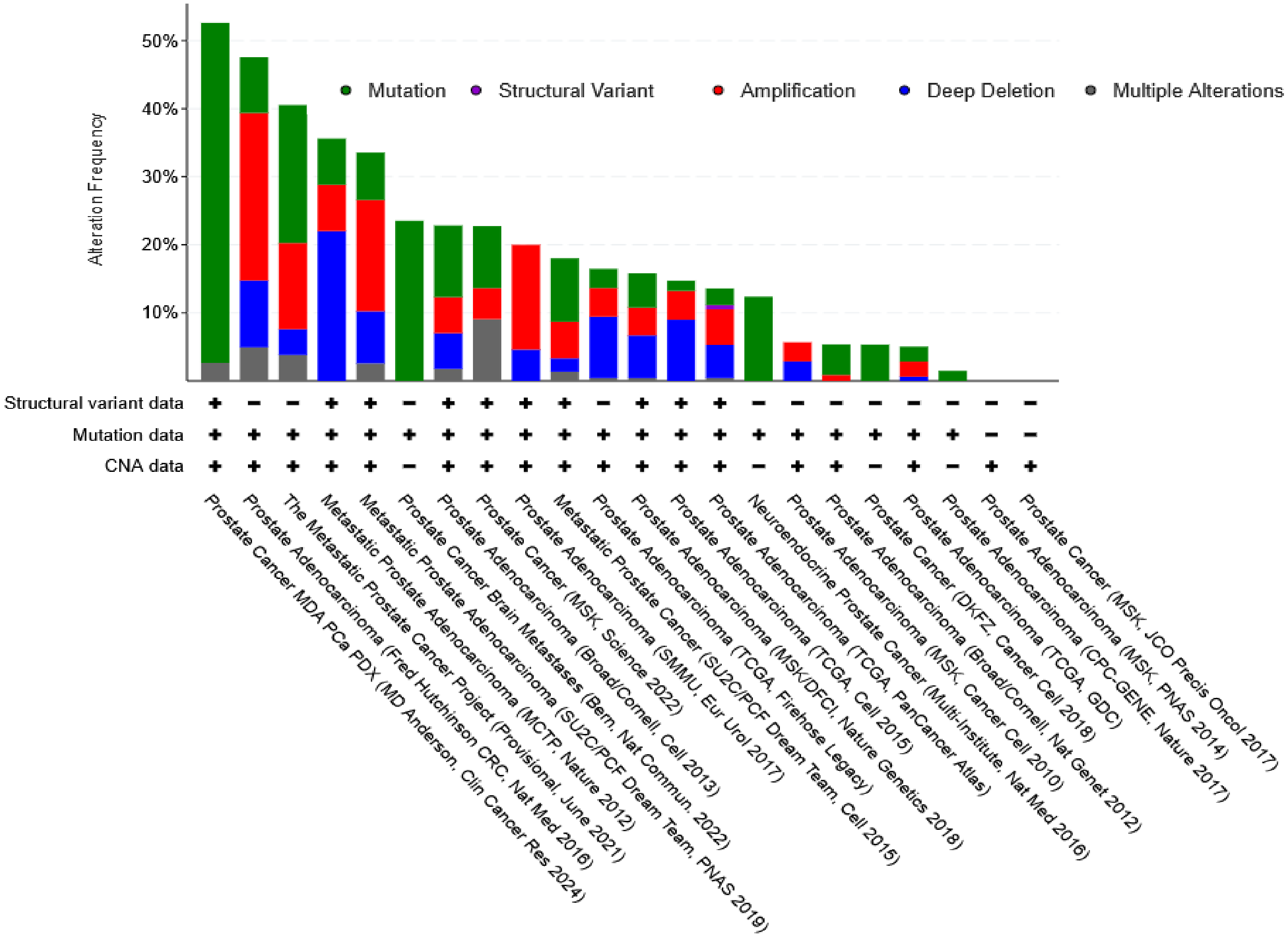

Supplemental Figure 2: Bar graph showing the frequency of genomic alterations, including gene amplifications, structural variants, deep deletions, and mutations, for ABC transporter genes across various prostate cancer patient cohorts available in cBioPortal from 13387 patients in 29 studies. Data Link: https://shorturl.at/zLHtd
